# LncRNA OTUD6B-AS1 promotes paclitaxel resistance in triple negative breast cancer by regulation of miR-26a-5p/MTDH pathway-mediated autophagy and genomic instability

**DOI:** 10.18632/aging.203672

**Published:** 2021-11-05

**Authors:** Peng-Ping Li, Rong-Guo Li, Yu-Qing Huang, Jin-Pian Lu, Wei-Jun Zhang, Zhen-Yu Wang

**Affiliations:** 1 Department of Breast-Thyroid Surgery, Department of General Surgery, The First Hospital of Xiaoshan District, Hangzhou, Zhejiang Province 311000, China; 2 The Second Affiliated Hospital of Zhejiang Chinese Medical University, Hangzhou, Zhejiang Province 310000, China

**Keywords:** chemotherapy resistance, autophagy, genomic instability (GIN), triple negative breast cancer (TNBC), DNA damage response (DDR)

## Abstract

Genomic instability (GIN) is pivotal in regulating tumor drug resistance, which blocked the treatment of triple negative breast cancer (TNBC). Although recent studies implied that non-coding RNA (ncRNA)-mediated autophagy abolishment promoted tumorigenesis by up-regulation of GIN, autophagy was known as a risk factor in tumor drug resistance. However, previous study also pointed that up-regulation of autophagy promoted GIN. Therefore, the relationship between autophagy and GIN is not clear, and more work is needed. And, if an ncRNA is identified to be a co-regulator of autophagy and GIN, it will be a potential therapy target of chemotherapy resistance in TNBC. In our study, we recognized both autophagy-GIN-associated microRNA (mi-26a-5p) by big data analysis, which was prognosis-correlated in breast cancer. Next, we identified the up-stream regulators (long non-coding RNA, lncRNA) and down-stream targets of miR-26a-5p by bioinformatics analysis (online public databases). Finally, we established lncRNA OTUD6B-AS1/miR-26a-5p/MTDH signaling pathway, and verified their functions by cytological, molecular biological and zoological experiments. In general, our study found (1) miR-26a-5p was a protective factor of breast cancer, while OTUD6B-AS1 and MTDH were risk factors; (2) OTUD6B-AS1 was the up-stream regulator of miR-26a-5p verified by luciferase; (3) up-regulation of miR-26a-5p and down-regulation of MTDH promoted cellular cytotoxicity of paclitaxel (PTX) *in vitro* and *in vivo*. (4) down-regulation of miR-26a-5p, overexpression of MTDH and OTUD6B-AS1 promoted autophagy and DNA damage; (5) up-regulation of OTUD6B-AS1 and MTDH inhibited DNA damage response (DDR) by inhibiting the phosphorylated activation of RAD51, ATR and ATM.

## INTRODUCTION

According to the Chinese Cancer Report 2019 and the World Health Organization (WHO) Global Cancer Report 2020, breast cancer is the highest incidence of malignant tumors in female and is one of the main causes of female malignant tumor death [1]. Due to the lack of HER-2 targeted therapy and endocrine therapy, paclitaxel (PTX)-based combination chemotherapy is still pivotal in triple-negative breast cancer (TNBC). However, up to 30%~50% chemotherapy resistance makes limited effects of combining drug treatment strategy, which lead bad prognosis [2]. Therefore, more work is needed to resolve chemotherapy resistance.

As we all know, genomic instability (GIN) is pivotal for tumor initiation and progression [3]. Amongst, DNA damage response (DDR) is an important process to maintain the genomic stability [3]. Tumor cells usually hold DDR defects and are prone to genetic alteration under the drug-induced microenvironment pressure, that is, gene copy number changes, chromosomal rearrangements, and gene mutations, which ultimately lead to tumor progression [3]. For example, in familial breast cancer, the direct loss, imbalance of expression and abnormal function of DDR protein (TP53, BRCA1, ATM, etc.) have led to an increased risk of breast cancer, the development of malignant subtypes, and tumor chemotherapy resistance [4].

Interestingly, autophagy is reported to be correlated to GIN. On the one hand, DDR-related protein promoted autophagy. For example, ATM activates autophagy by AMPK/TSC2/mTORC1 pathway [5]. Besides, ATM directly phosphorylated and stabilized nuclear TP53 to promote autophagy. On the other hand, molecular chaperone-associated autophagy (CMA) maintained the stability of the MRN complex by directly or indirectly regulating the level of CHEK1, thereby promoting DDR [6]. In addition, autophagy regulated the stability of FLNA and RAD51 in the nucleus by controlling the protein level of p62/SQSTM1, thereby promoting the production of non-homologous end binding (NHEJ) [7, 8]. That means, autophagy inhibits tumor chemotherapy resistance, and abolishment of autophagy may promote tumor chemotherapy resistance by increasing GIN. It seems to provide a reasonable explanation for the failure of autophagy-targeted therapy in clinical trials. In fact, previous study suggested that non-coding RNA (ncRNA) can promote GIN by up-regulating autophagy, and it is caused by the up-regulation of Ros [9]. However, autophagy is also an inhibitor of DDR. Previous study showed that the autophagy activator rapamycin significantly inhibited HR and NHEJ to promote GIN after radiotherapy [10]. Therefore, the relationship between autophagy and DDR is still unclear. And work about the roles of ncRNA in GIN-mediated autophagy-targeted therapy failure will be helpful to develop the efficient autophagy-targeted therapy.

In fact, in many studies, it has been suggested that ncRNA can regulate DDR-related proteins and participate in tumor regulation. For example, in head and neck squamous cell carcinoma, miR-205-5p down-regulates BRCA1 expression, inhibits DDR, promotes cell growth and tumor metastasis [11]; in osteosarcoma cells and cervical cancer cells, miR-22 inhibits the DDR process by targeting MDC1, Increase cell radiotherapy tolerance; in glioblastoma, miR-1193 directly targets YY1AP1 (YY1-associated protein 1), thereby inhibiting FEN1 (Flap endonuclease 1), leading to the accumulation of DNA double-strand breaks, thereby increasing Genomic instability [12]. Therefore, there is likely to find an ncRNA which hold same effects on autophagy and GIN, and it may resolve the autophagy-targeted therapy failure or limited effects in present clinical trials. And it may give a new strategy for resolving chemotherapy resistance in TNBC.

In this paper, we identified miR-26a-5p is an autophagy-related, GIN-related, and prognosis-related ncRNA in breast cancer through big data analysis from online public database. And by bioinformatics analysis and molecular biological experiments, we pointed that lncRNA OTUD6B-AS1 was an up-stream of miR-26a-5p, while MTDH was a down-stream target of miR-26a-5p. Finally, we established OTUD6B-AS1/miR-26a-5p/MTDH pathway for regulation both of autophagy and GIN in TNBC.

## MATERIALS AND METHODS

### Online data

#### Data collection

Protein and lncRNAs were extracted from transcriptional profile (RNA sequence data, from The Cancer Genome Atlas, TCGA: https://portal.gdc.cancer.gov/) with the help of Perl Strawberry 5.3; miRNAs were collected from transcriptional profile (isoform expression quantification data, from TCGA); 144 DDR-associated genes were collected from literature review, and the expression profile was extracted from transcriptional profile (RNA sequence data, from TCGA) with the help of Perl Strawberry 5.3; miRNA targeted genes were identified by combination analysis from miRDB, miRTarBase and TargetScan database, with the help of Perl Strawberry 5.3; Autophagy-associated genes were identified by Human Autophagy Database (HADb: http://www.autophagy.lu/), and the expression profile was extracted from transcriptional profile (RNA sequence data, from TCGA) with the help of Perl Strawberry 5.3; Clinical data was collected from cBioportal (http://www.cbioportal.org). miR-26a-5p-interacted lncRNAs were collected from LncBase 2.0 (http://carolina.imis.athena-innovation.gr/diana_tools/web/index.php). Pan-cancer analysis of MTDH (AGE-1) was performed in Gene Expression Profiling Interactive Analysis (GEPIA, http://gepia.cancer-pku.cn/). Partial prognosis data were collected from Kaplan-Meier Plotter (K-MPlotter, http://kmplot.com/analysis/index.php?p=background).

#### Analysis

Prognosis-related miRNAs and lncRNAs were identified by Univariate Cox Regression Analysis in R 4.0.5. The survival curve was made by Kaplan-Meier (K-M) analysis in SPSS 20.0. The Risk model was made by Multivariate Cox Regression Analysis, and the receiver operating characteristic curve (ROC curve) was performed by packages (survivalROC) in R4.0.5. Nomogram was performed by R4.0.5. Differential genes were identified by R4.0.5. Co-expression analysis and correlative analysis were performed by Pearson Test in R4.0.5. KEGG and Go pathway analysis were mainly performed in R4.0.5 by packages (clusterProfiler, org.Hs.eg.db, enrichplot, and ggplot2). The difference of genomic altered fraction between TNBC and other types of breast cancer was analyzed by Chi-square Test in SPSS 20.0.

### Cellular experiments

#### Reagents and drugs

Paclitaxel was purchased from Shelleck (Cat. No: S1150, Shanghai, China), Rapamycin (RAPA, Cat. No: HY-10219, MedChemExpress) was purchased from MedChemExpress (China). Bovine serum albumin (BSA) was purchased from Sigma-Aldrich. Puromycin was purchased from Zorin Biology Co., Ltd (Shanghai, China).

#### Breast cell lines and culture

Breast cancer cell lines (MDA-MB-231 and HCC1937) were purchased from Procell Life Science and Technology Co., Ltd (Wuhan, China) in 2020 with STR matching analysis. MDA-MB-231 was cultured in DMEM (Gibco, USA), HCC1937 was cultured in 1640 (Gibco, USA). All types of culture media were supplemented with 10% fetal calf serum (Biological Industries, BI, USA) and 100 units/mL penicillin and streptomycin. OPTI-MEM was purchased from Gibco (USA).

#### Cell proliferation and cytotoxicity assays

The protocol for the detection of cell viability is as same as our previous work [PMID: 33282725] [13]. Simply, the standardized curve was firstly established: optical density (OD) of (0.1, 0.2, 0.4, 0.8, 1.0, 1.5, 2.0, 3.0) × 10^5^ cells were detected after 3 to 4 hours of treatment of CCK8 (YEASEN Biotech Co., Ltd, China) after being transplanted into 96-wells plates, then the linear standard curve between log [cell quantity] and OD was fit. Cells were plated in 96-wells plates, and different treatments were performed followed by 3 to 4 hours treatment of CCK8. Next, the OD value of those cells was analyzed in the above standardized curve.

#### Quantitative real-time PCR (qRT-PCR)

The protocol of qRT-PCR was shown in our previous study [PMID: 31935687]. Simply, the Trizol RNA isolation system (Invitrogen, USA) was used for total RNA extraction. The cDNA templates were synthesized through PrimeScript RT Reagent Kit (TaKaRa, China), and qRT-PCR was performed with a 7500 Fast™ System (Applied Biosystems, USA) using the Sensi Mix SYBR Kit (Bio-Rad, USA). The mRNA level was calculated via using (=2−ΔΔCt) and normalized to GAPDH. All of the sequences of primer were designed by Primer 5 soft.

#### Western blot analysis

Cells were harvested by cytology brush and lysed with RIPA lysis buffer (YEASEN Biotech Co., Ltd, China) supplemented with phosphorylase and protease inhibitor mixture (YEASEN Biotech Co., Ltd, China), quantified by the BCA assay. The standard detail experimental process of the western blot was the same as our previous study (PMID: 33282725). Western blot band was quantified through the Image-J software (NIH, USA). Antibodies against ATM (Ab-AF4119# #647), Ser1981 phosphorylated ATM (p-ATM, Ab-AF-4129# #647), ATR (Ab-DF2631# #647), Ser428 p-ATR (Ab-DF7512# #647), RAD52 (aB-df7175# #647), Tyr104 p-RAD52 (Ab-AF4431# #647) were purchased from Affinity Bioscience (1:500, Wuhan, China); Antibodies against MTDH (1:1000, 13860-1-AP), E-cadherin (1:1000, 20874-1-AP), vimentin (1:1000, 10366-1-AP), snail1 (1:500, 13099-1-AP), twist1 (1:1000, 25465-1-AP) and ZO-1 (1:2000, 21773-1-AP) were purchased from Protein Technology (Wuhan, china); Antibody against GAPDH was purchased from YEASEN (1:5000, 30202ES40, Shanghai, China); Antibody against γ-H2AX was purchased from Abcam (1:1000, ab81299, China), antibodies against.

#### Immunofluorescence analysis

Briefly, breast cancer cells (MDA-MB-231 and HCC1937) were seeded in 12-well plates for 24 h, followed with or without different treatments. Then, cells were fixed with 4% paraformaldehyde, permeabilized by 0.5% Triton X-100, and blocked with 5% bovine serum albumin (BSA, Sigma) for 1 h at 37°C. Next, the above cells were incubated with primary antibodies (γ-H2AX, 1:100) overnight at 4°C. Subsequently, they were washed by PBS and incubated with secondary antibodies for 1h at room temperature before being washed again. Finally, nuclei were stained with 5 μL DAPI (Haotian Biology, Co., Ltd, Hangzhou, China) before being detected by a fluorescence microscope.

#### Alive and death cells staining

The Alive and Death cells staining was carried out using Calcein AM/PI staining assay (YEASEN Biotech Co., Ltd, China). After being seeded in a 24-well plate and cultured for 24 h, breast cancer cells were treated with different treatments. Then all cells were co-cultured with Calcein AM and PI for 30 mins in 37°C, followed by being observed in fluorescence microscope.

#### Transfection of recombination plasmid (RP) and small interfere RNA (siRNA)

The OTUD6B-AS1-WT (wide type) and OTUDB6-MUT (mutation type) contained recombination plasmid were synthesized by Shanghai Kecong Biology and Science Co., Ltd. The sequence of OTUD6B-AS1-WT is “AACAATAAAGGATCTACTTGAAA”, and the sequence of OTUD6B-AS1-MUT is “AACAATAAAGGATCGCAGGTCC”. 5 × 10^−5^ cells were transplanted into 6 wells plates for 24 h, and then transfected with RP or siRNA for 48 h with Hieff Trans™ Liposomal Transfection Reagent (YEASEN Biotech Co., Ltd, China) for the best transfection efficiency, according to the manufacturer’s instructions. All sequences of mimic, inhibitor, siRNAs were displayed in Supplementary Table 1.

#### PTX resistant cell lines establishment

Simply, the IC50-1 was firstly detected by CCK8 according to the above protocol. Then, 2 × 10^−6^ cells (MDA-MB-231 and hcc1937) were planted in fr25 cm2 plates for 24 h, which followed by 48 h-treatment of 1/8 IC50 PTX (with or without other drugs). Next, replaced fresh culture for proliferation to about 2 × 10^−6^ cells. Repeated the above operation 5 times and measured IC50 again. Until the IC50 doubles, the cell lines were defined as PTX-resistant cell lines. All works of this part with the help of Shanghai Kecong Biology and Science Co., Ltd.

#### GFP-RFP-LC3 dual fluorescent-labeled cell lines establishment

The stubRFP-sensGFP-LC3 lentivirus was synthesized by Zorin Biology Co., Ltd (Shanghai, China). 5 × 10^−5^ cells were panted in 6-wells plates for 24h and followed by 48 h-treatment of 2 ml OPTI-MEM, which containing 5 μg/ml polybrene and 5 × 10^−8^ stubRFP-sensGFP-LC3 lentivirus. Then, replaced fresh culture for another 48 h. The IC50 of puromycin in HCC1937-WT, MDA-MB-231-WT, HCC1937- GFP-RFP-LC3, and MDA-MB-231-GFP-RFP-LC3 were detected by CCK8. Finally, 2 μg/ml puromycin was used to maintain GFP-RFP-LC3 expression, and 4 μg/ml puromycin was used to screen GFP-RFP-LC3 labeled cells.

#### Luciferase assay

This part of the experiment was performed by Shanghai Kecong Biology and Science Co., Ltd. The sequence of miR-26a-5p is “UUCAAGUAAUCCAGGAUAGGCU”.

#### Migration assay

Migration ability was detected by trans-well. For trans-well, 50000 cells, with special treatments or not, were transplanted into trans-well plates (24-well, 8.0 μm, Corning Incorporated, Corning, NY, USA) with a 10% gradient of fetal calf serum for 48 h. The detection procedure was the same as our previous study (PMID31935687). Quantification of passed cell area was performed by Image-ProR Plus.

### Animal experiments

Eight 4–6 weeks female nude mice were injected subcutaneously with 1 × 10^−6^ cells (HCC1937), which was followed by peritumoral injection of liposome-encapsulated miR-26a-5p-inhibitor or miR-con, with veil tail-vein injection of paclitaxel (the details were displayed in Supplementary Figure 1).

### Clinical samples collection

This study was approved by The First Affiliated Hospital of Anhui Medical University Review Board and the ethics committees of Anhui Medical University. 7 paraffin-embedded tissue sections and clinical frozen tissue samples were collected from a tissue bank from January 2008 to January 2011. All patients with breast cancer were confirmed by at least two pathologists.

#### Immunohistochemistry staining and scoring standard

Experiment’s procedure of immunohistochemistry for MTDH expression level were performed as previously described (PMID: 31935687). What more, the work concentration of antibody against MTDH (Protein technology, Wuhan, China) was 1:100. The protein expression level was assessed by Mean of Integrated Option Density (IOD) with Image-ProR Plus. Briefly, all of the Immunohistochemical sections were photographed for three yields in the same standard, and then select Area of Interesting (AOI) and detect IOD to gain Mean of IOD (IOD/AOI, MI).

### Statistics

All experimental data were presented as the means ± SD. Statistical Package for the Social Sciences version 20.0 (SPSS Inc., USA) was used for statistical analyses. ANOVA, paired *t*-test, Chi-square (χ^2^) test, and nonparametric test (Mann Whitney U) were used for statistical analysis of different situations. Statistical significance was considered when *p* < 0.05 (^*^p < 0.05; ^**^p < 0.01; ^***^p < 0.001; ns: *p* > 0.05). All histograms and curves were constructed with GraphPad Prism 8.0 software (GraphPad Software, La Jolla, CA, USA). All experiments were repeated at least three times to gain reliable data.

### Ethics statement

The studies involving human participants were reviewed and approved by The First Affiliated Hospital of Anhui Medical University Review Board and the ethics committees of Anhui Medical University. The animal study was reviewed and approved by The First Affiliated Hospital of Anhui Medical University Review Board and the ethics committees of Anhui Medical University.

### Data availability statement

All data of this paper was display in manuscript, and raw data could be got from corresponding authors and journal.

## RESULTS

### Recognition of prognosis-related miRNAs which was involved in autophagy and GIN

**Figure 1:**
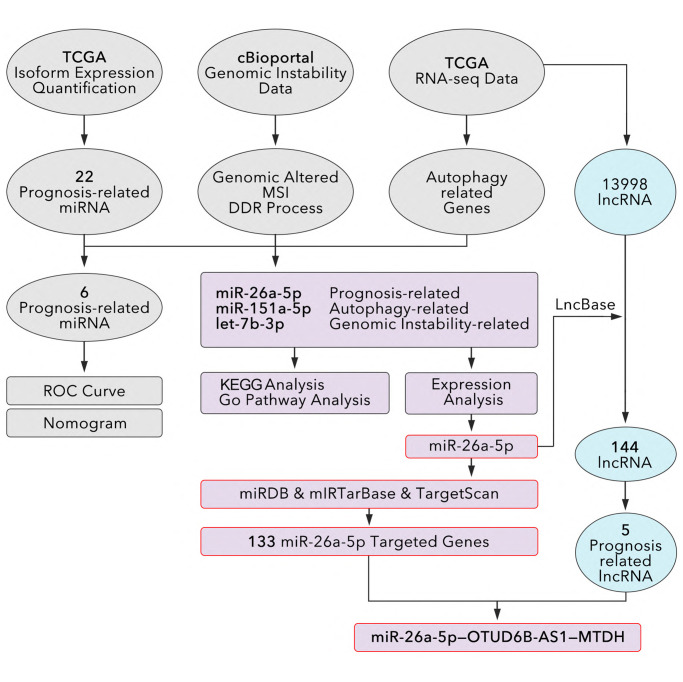
**The study design to identify an autophagy-related and genomic-instability-related lncRNA-miRNA-Gene pathway in breast cancer.** mRNAs and lncRNAs are identified from RNA-seq data. the Human Autophagy Database (HADb: http://www.autophagy.lu) is used to identify autophagy-related genes. Univariate and multivariate Cox regressions are used to identified prognosis-related miRNA, and co-expression is used to identify prognosis-related miRNA. miRDB, miRTarBase, and TargetScan are used to recognize miRNA-targeted genes. LncBase 2.0 is used to identify lncRNAs that interact with miR-26a-5p. Co-expression analysis between lncRNA-genes and miRNA-genes is performed to identified lncRNA-miRNA-gene pathways.

**Figure 2:**
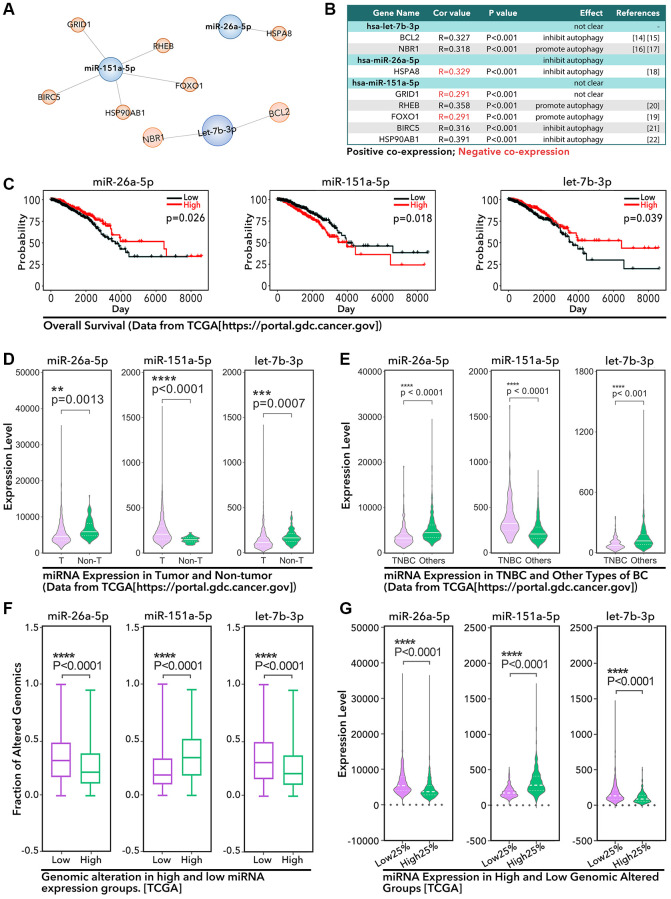
**The identified 3 miRNAs in breast cancer.** (**A**) Three autophagy-related miRNAs are identified, among which (**B**) let-7b-3p is positive co-expression with BCL2 and NBR1, miR-26a-5p is negative co-expression with HSPA8, miR-151a-5p is positive co-expression with RHEB, BIRC5, and HSP90AB1, and negative co-expression with GRID1 and FOXO1. (**C**) The prognosis characteristics of three miRNAs from TCGA by K-M analysis. (**D**) The expression of 3 miRNAs in breast cancer tissues and adjacent tissues. (**E**) The expression of 3 miRNAs in TNBC tissues and other types of breast cancer tissues. (**F** and **G**) The relationship between the expression profile of 3 miRNAs and the genomic altered faction status.

The research process was showed in Figure 1, and the details are displayed in the following: our study identified 706 miRNAs in breast cancer from TCGA database, among which 22 miRNAs were prognosis-related identified by Cox Regression made by R studio, and the details were shown in Supplementary Table 2. Next, we performed Multivariate Cox Regression and made a risk model based on the above 22 prognosis-related miRNAs. However, combining with clinical data (from TCGA and cBioportal), we found it was not powerful to make prognosis prediction based on available evidence in the ROC curve and nomogram (Supplementary Figure 1). Then, we extracted autophagy associated genes in the help of HADb online database, and the combining analysis found miR-26a-5p, miR-151a-5p, and let-7b-5p were autophagy-associated miRNAs. Finally, we identified all of those three miRNAs were GIN-related miRNAs. As showed in Figure 2A and 2B, let-7b-3p was positive co-expression with BCL2 (R = 0.327, *P* < 0.001) and NBR1 (R = 0.318, *P* < 0.001), among which the BCL2 was reported to inhibit autophagy [14, 15], while NBR1 was reported to promote autophagy [16, 17]. miR-26a-5p and HSP-A8 were negative co-expression (R = −0.329, *P* < 0.0001), which was an autophagy inhibitor [18]; miR-151a-5p and GRID1 (R = −0.291, *P* < 0.0001) and FOXO1 (R = −0.291, *p* < 0.0001) were negative co-expression, amongst which FOXO1 was reported as an autophagy promotor [19]; while miR-151a-5p was positive co-expression with RHEB (R = 0.358, *p* < 0.0001), BIRC5 (R = 0.316, *p* < 0.0001) and HSP90AB1 (R = 0.391, *p* < 0.0001), among which RHEB was reported as an autophagy promotor [20], while BIRC5 and HSP90AB1 were reported as autophagy inhibitor [21, 22]. K-M analysis from TCGA data showed miR-26a-5p (*p* = 0.026, Figure 2C) and let-7b-3p (*p* = 0.039, Figure 2C) were protective factors, while miR-151a-5p (*p* = 0.018, Figure 2C) was a risk factor for breast cancer. And then, we explored the expression level of the above 3 miRNAs. As the results showed, miR-26a-5p (*p* = 0.0013, Figure 2D) and let-7b-3p (*p* = 0.0007, Figure 2D) were lower expression in tumor tissues, while miR-151a-5p (*p* < 0.0001, Figure 2D) was higher expression in tumor tissues, as compared to non-tumor tissues. Meantime, miR-26a-5p (*p* < 0.0001, Figure 2E) and let-7b-3p (*p* < 0.001, Figure 2E) were lower expression in TNBC, while miR-151a-5p (*p* < 0.0001, Figure 2E) was higher expression in TNBC, as compared to other types of breast cancer. To verify the effects of 3 miRNAs in GIN, we made further analysis. As Figure 2F showed, lower expression of let-7b-3p and miR-26a-5p accompanied with a higher level of genomic altered fraction (*p* < 0.0001, Figure 2F), while lower expression of miR-151a-5p accompanied with a lower level of genomic altered fraction (*p* < 0.0001, Figure 2F). Meantime, higher level of genomic altered fraction group held lower expression level of miR-26a-5p and let-7b-3p (*p* < 0.0001, Figure 2G), but higher expression level of miR-151a-5p (*p* < 0.0001, Figure 2G).

### KEGG and go analysis

**Figure 3:**
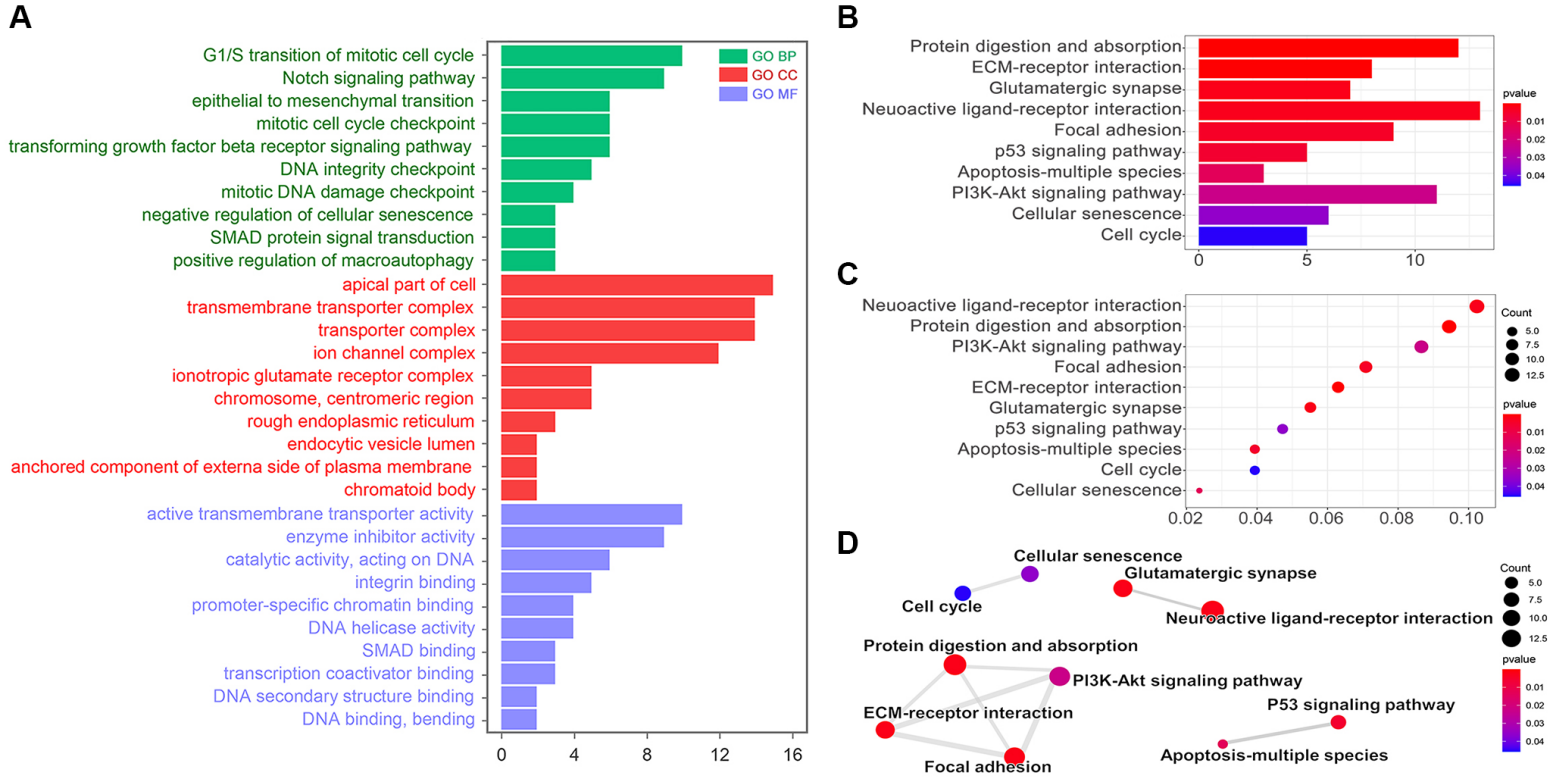
**KEGG and GO analysis.** (**A**) Go pathway (Biological Process, BP; Cellular Component, CC; Molecular Function, MF) analysis by R. KEGG analysis results showed by (**B**) bar diagram, (**C**) bubble diagram, and (**D**) interaction diagram.

We made a risk model based on those 3 miRNAs by R4.0.5. According to the risk score, we grouped TCGA data to two groups. And we found out 233 differential genes. Go analysis showed those differential genes were involved in the cell cycle (G1/S transition of the mitotic cell cycle) and cell cycle checkpoint (mitotic cell cycle checkpoint, DNA integrity checkpoint, and mitotic DNA damage checkpoint) (*p* < 0.05, Figure 3A). Meantime, the KEGG analysis showed that those differential genes were involved in the regulation of focal adhesion, p53 signaling pathway, apoptosis-multiple species, PI3K-AKT signaling pathway, cellular senescence, and cell cycle (*p* < 0.05, Figure 3B and 3C). The interaction analysis showed that the cell cycle was related to cellular senescence; PI3K-AKT signaling pathway was related to protein digestion and absorption, ECM-receptor interaction and focal adhesion; p53 signaling pathway was related to apoptosis-multiple species (Figure 3D). As we all known, cell cycle checkpoints were important in DNA repair and maintaining genomic stability, while p53 signaling pathway and PI3K-AKT signaling pathway were important in regulation of autophagy. Therefore, miR-26a-5p, miR-151a-5p, and let-7b-5p were further considered to be involved in autophagy and GIN.

### miR-26a-5p/MTDH pathway was identified as a potential regulator of chemotherapy resistance in TNBC which was regulated by lncRNA OTUD6B-AS1

**Figure 4:**
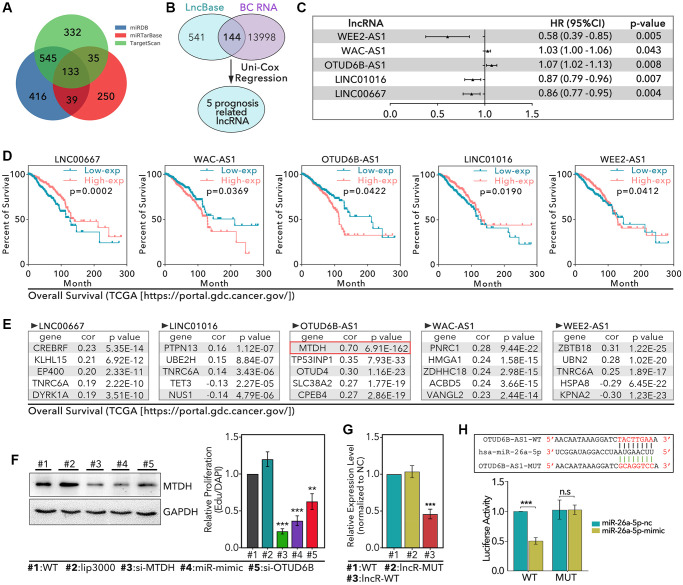
**Identification of OTUD6B-AS1-miR-26a-5p-MTDH signaling pathway.** (**A**) miRDB, miRTarBase, and TargetScan databases are used to identify 133 miR-26a-5p targeted genes. (**B**) 541 lncRNAs are collected from The LncBase 2.0 which interacts with miR-26a-5p, among which 144 lncRNAs are found in 13998 breast cancer-associated lncRNAs; 5 prognosis-related lncRNAs is identified. (**C**) The prognosis risk of 5 lncRNAs in breast cancer. (**D**) K-M analysis of 5 lncRNAs, data from TCGA. (**E**) The top5 genes with the most strength correlation of 5 lncRNAs. (**F**) WB assay to detect the level of MTDH in HCC1937 after different treatments. (**G**) RT-qPCR to detect the level of miR-26a-5p. (**H**) Luciferase to detect the interaction between miR-26a-5p and OTUD6B-AS1.

Basing on literature review, we found that miR-26a-5p was exactly a regulator of autophagy in previous studies, such as miR-26a-5p interfered autophagy process by regulation of ULK1/2, smad1 and ATG12 [23–26]. In addition, miR-26a-5p was reported to regulate expression of DAPK1, by which it can interfere the autophagy process [27]. In fact, DAPK1 as a down-stream target of p53, was an important role in p53-mediated DNA damage repair under genotoxic stress microenvironment [28]. Meantime, as a reported target, miR-26a-5p was involved in tumor cell proliferation and invasion in breast cancer [29]. However, the roles of miR-26a-5p in autophagy-mediated and genomic-instability-mediated chemotherapy resistance in TNBC were not clear. Therefore, our study chose the miR-26a-5p into our further exploration. First, we identified 133 down-stream targets of miR-26a-5p (Figure 4A and Supplementary Figure 2). In addition, lncRNA usually down-regulated level of miRNA. Therefore, in here, we identified 5 miR-26a-5p-interacted and prognosis-correlated lncRNAs, which potentially inhibited the level of miR-26a-5p (Figure 4B and 4C). The process of identifying miR-26a-5p-interacted and prognosis-correlated lncRNAs was showed in Figure 1, and simple description was that our study extracted 13998 lncRNAs from TCGA database in breast tissues, 144 of which were prognosis-associated. Then, we extracted 650 miR-26a-5p-interacted lncRNAs from lncBase2.0 database. Finally, we identified 5 miR-26a-5p-interacted and prognosis-associated genes by combining analysis. As results showed, WEE2-AS1 (0.58 [0.39–0.85], HR [95%CI], *p* = 0.005), LNC01016 (0.87 [0.79–0.96], *p* = 0.007) and LINC00667 (0.86 [0.787–0.95], *p* = 0.004) were protective factors of breast cancer, but they were not reasonable in promoting tumor progression by down-regulation of miR-26a-5p, for which miR-26a-5p was a tumor inhibitor. Besides, WAC-AS1 (1.03 [1.00–1.06], *p* = 0.043) and OTUD6B-AS1 (1.07 [1.02–1.13], *p* = 0.008) were risk factors of breast cancer, which implied they potentially down-regulated the level of miR-26a-5p to promote tumor progression (Figure 4C). The same results were displayed in K-M analysis (Figure 4D). Following, we identified OTUD6B-AS1-correlated and WAC-AS1-correlated genes, which were also the targets of miR-26a-5p (Figure 4E; we just list the top10 correlative genes). As Figure 4E showed, all correlation coefficients between lncRNAs and genes were lower than 0.4, except MTDH (R = 0.7, *p* = 6.91e-162), which mean MTDH was most likely the down-stream target both of miR-26a-5p and OTUD6B-AS1. Up to now, we originally established OTUD6B-AS1/miR-26a-5p/MTDH signaling pathway. In order to verify the above pathway, our study performed following experiments. As the results showed, miR-26a-mimic decreased the expression level of MTDH more than 50% (*p* < 0.001, Figure 4F), and si-OTUD6B-AS1 decreased the expression level of MTDH about 40% (*p* < 0.01, Figure 4F). Meantime, we found OTUD6B-AS1-WT (lncR-WT) decreased the level of miR-26a-5p about 50% (*P* < 0.001, Figure 4G), and the luciferase assay showed that OTUD6B-AS1-WT decreased the luciferase activity of miR-26a-5p-mimic, while OTUD6B-AS1-MUT rarely made no effect on it (*p* < 0.001, Figure 4H).

### MTDH was a risk factor in breast cancer

**Figure 5:**
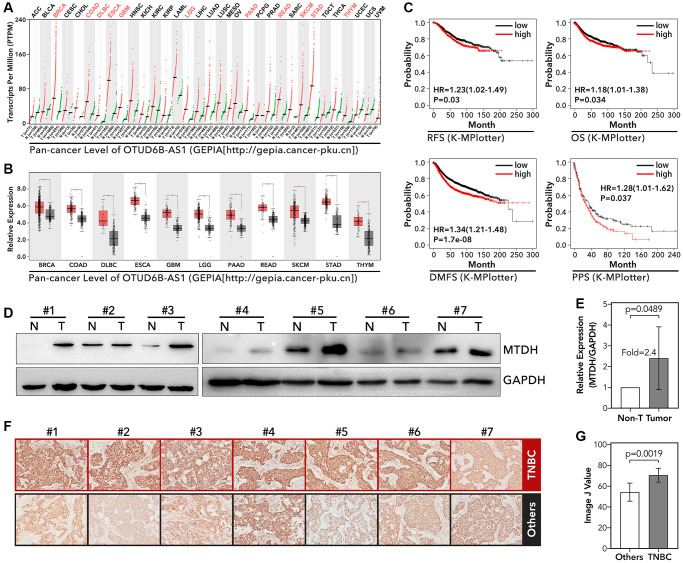
**The expression level of MTDH and its molecular function in tumor.** (**A** and **B**) Pan-cancer analysis about the expression level of MTDH, data from GEPIA (http://gepia.cancer-pku.cn). (**C**) The recurrence-free survival (RFS), overall survival (OS), disease metastasis-free survival (DMFS), and post-operation progressive survival (PPS) of MTDH in breast cancer, data from K-MPlotter (http://kmplot.com/analysis/index.php?p=background). (**D** and **E**) The expression level of MTDH in frozen TNBC tissues and adjacent tissues, detected by WB. (**F** and **G**) The expression level of MTDH in TNBC and other types of breast cancer, detected by IHC.

To our knowledge, MTDH has been reported that its overexpression promoted PTX resistance, tamoxifen resistance, and doxorubicin resistance in luminal-A and TNBC breast cancer [30–32], and MTDH-based DNA vaccine suppressed lung metastasis and enhanced chemosensitivity to doxorubicin in breast cancer [33]. And in our study, we found that MTDH was an abnormally higher expression in BRCA, COAD, DLBC, ESCA, GBM, LGG, PAAD, READ, SKCM, STAD, and THTM by pan-cancer analysis (*p* < 0.001, Figure 5A and 5B). K-M analysis showed that higher expression level of MTDH accompanied with worse relapse-free survival (RFS, 1.23 [1.02–1.49], *p* = 0.03, Figure 5C), worse overall survival (OS, 1.18 [1.01–1.38], *p* = 0.034, Figure 5C), worse disease metastasis-free survival (DMFS, 1.34 [1.21–1.48], *p* = 1.7E-8, Figure 5C) and worse post-operative progression survival (PPS, 1.28 [1.01–1.62], *p* = 0.037, Figure 5C) in breast cancer. Furthermore, we detected the expression level of MTDH in TNBC and normal tissues. As the Figure 5D and 5E showed, TNBC tissues held higher level of MTDH than normal tissues (fold = 2.4, *p* = 0.0489). Meantime, the Figure 5F and 5G showed that TNBC held higher level of MTDH than other types of breast cancer (*p* = 0.0019).

### miR-26a-5p/MTDH pathway regulated PTX resistance *in vitro* and *in vivo* in TNBC

**Figure 6:**
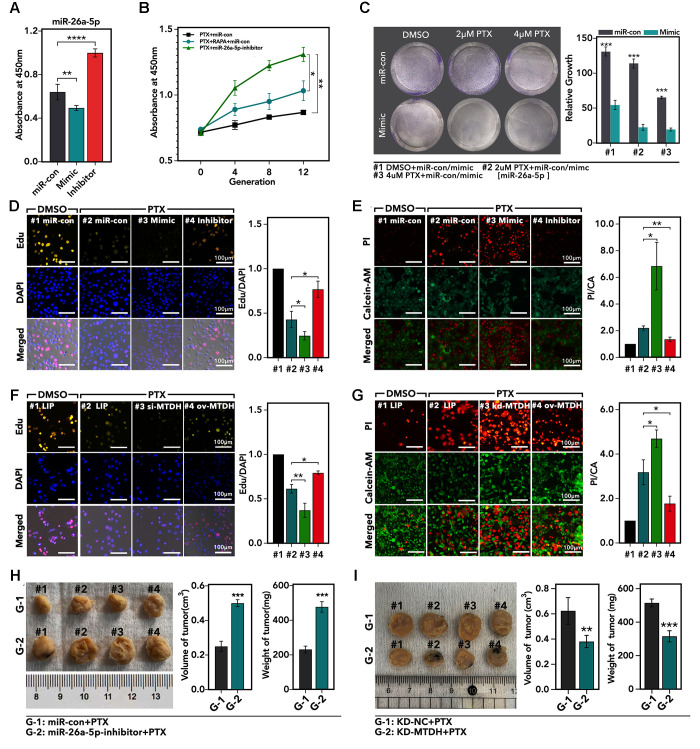
**The effects of miR-26a-5p/MTDH pathway on regulation of PTX resistance.** (**A**) The relative cell viability of HCC1937 cells, which are treated with miR-mimic, miR-inhibitor, or miR-con for 24 h followed by 48 h-treatment of PTX, detected by CCK8 assay. (**B**) The absorbance of different generations of PTX-resistance HCC1937 is treated with 48 h-treatment of paclitaxel, detected by CCK8 assay. (**C**) Clone formation of HCC1937, which are treated with different treatments. (**D**) The proliferation of HCC1937, which are treated with miR-mimic, miR-inhibitor, or miR-con for 24 h followed by 48 h-treatment of PTX, detected by Edu assay. (**E**) The cellular viability of HCC1937, which are treated with miR-mimic, miR-inhibitor, or miR-con for 24 h followed by 48h-treatment of PTX detected by Alive/Dead assay. (**F**) The proliferation of HCC1937, which are treated with liposome, si-MTDH, or ov-MTDH (MTDH re-combination plasmid) for 24 h followed by 48 h-treatment of PTX, detected by Edu assay; (**G**) The cellular viability of HCC1937, which are treated with liposome, si-MTDH, or ov-MTDH for 24 h followed by 48 h-treatment of PTX detected by Alive/Dead assay. (**H** and **I**) Subcutaneously transplantation tumor model.

As the results showed, the miR-26a-5p-inhibitor increased cell viability about 0.5-fold which treated with PTX (*p* < 0.0001, Figure 6A), while miR-26a-5p-mimic inhibited cell viability about 20% (*p* < 0.01, Figure 6A), as compared to miR-con group. As Figure 6B showed, miR-26a-5p-inhibitor was more powerful in promoting PTX resistance formation as compared with single PTX treatment (*p* < 0.01, Figure 6B) or PTX + RAPA combining treatment (*p* < 0.05, Figure 6B). In clone formation assay, we found that miR-26a-5p-mimic significantly promoted the sensitivity of HCC1937 to PTX treatment (*p* < 0.001, Figure 6C). And the Edu assay showed that miR-26a-5p-inhibitor increased the ratio of Edu/DAPI about 1.4-fold, while miR-26a-5p-mimic decreased ratio about 35% (*p* < 0.05, Figure 6D). In addition, we performed Alive/Dead assay, and the showed that miR-26a-5p-mimic increased the fraction of dead cells about 3-fold (*p* < 0.001, Figure 6E), while miR-26a-5p-inhibitor decreased the fraction of dead cells about 30% (*p* < 0.05, Figure 6E), as compared with miR-con group. On contrary, down-regulation of MTDH decreased the ratio of Edu/DAPI about 37% (*p* < 0.001, Figure 6F), while up-regulation of MTDH increased the ratio about 0.25-fold (*p* < 0.05, Figure 6F). Besides, our study found same effects of MTDH in defending against PTX-mediated cell death: down-regulation of MTDH increased the PTX-mediated cell death about 30% (*p* < 0.05, Figure 6G), while up-regulation of MTDH decreased the PTX-mediated cell death about 45% (*p* < 0.05, Figure 6G). Furthermore, we performed subcutaneous tumor model to verify the effect of miR-26a-5p and MTDH in PTX resistance. As the results showed, peritumoral injection of liposome-encapsulated miR-26a-5p-inhibitor promoted subcutaneous tumor growth about 50% under treatment of PTX (*p* < 0.001, Figure 6H), and down-regulation of MTDH increased the PTX-induced tumor growth inhibition about 30% (*p* < 0.001, Figure 6I).

### OTUD6B-AS1/miR-26a-5p/MTDH pathway regulated autophagy and DDR process

**Figure 7:**
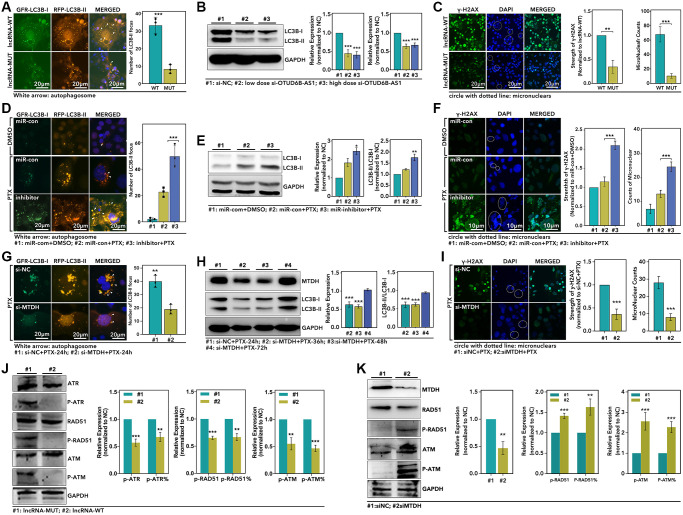
**The OTUD6B-AS1-miR-26a-5p-MTDH pathway mediated autophagy and DDR process.** (**A**) IF assay displays the LC3B-I and LC3B-II focus in HCC1937 (green: LC3B-I; orange: LC3B-II; blue: DAPI), (**B**) WB assay detects the level of LC3B-I, LC3B-II and MTDH, (**C**) and IF assay displays the expression level of γ-H2AX (Green: γ-H2AX; Blue: DAPI), which are treated with recombined plasmid lncRAN-MUT or lncRNA-WT; (**D**) IF assay displays the LC3B-I and LC3B-II focus in HCC1937, (**E**) WB assay detects the level of LC3B-I, LC3B-II and MTDH, (**F**) and IF assay displays the expression level of γ-H2AX, which are treated with different combinations with in miRNA and PTX; (**G**) IF assay displays the LC3B-I and LC3B-II focus in HCC1937, (**H**) WB assay detects the level of LC3B-I, LC3B-II and MTDH, (**I**) and IF assay displays the expression level of γ-H2AX, which are treated with si-NC+PTX or si-MTDH+PTX; (**J** and **K**) WB assay detect the level of DDR process associated markers (p-ATR, ATR, p-ATM, ATM, p-RAD51, RAD51) after different treatments in HCC1937.

As the results showed, over-expression of OTUD6B-AS1 promoted autophagy process by up-regulation of the LC3B-II counts about 3-fold (*p* < 0.001, Figure 7A), and the WB displayed down-regulation of OTUD6B-AS1 decreased the expression level of LC3B-II and the ratio of LC3B-II/LC3B-I more than 50% (*p* < 0.0001, Figure 7B). As for GIN, we found that over-expression level of OTUD6B-AS1 increased the γ-H2AX about 2-fold (*p* < 0.001, Figure 7C), and increased the micronuclear counts about 5-fold (*p* < 0.0001, Figure 7C). Following, we explored the effect of miR-26a-5p in autophagy and GIN. As the results showed, miR-26a-5p-inhibitor significantly increased the number of LC3B-II focus about 2-fold (*p* < 0.001, Figure 7D), and the WB assay showed that miR-26a-5p-inhibitor up-regulated the expression of LC3B-II and the ratio of LC3B-II/LC3B-I more than 1.5-fold (*p* < 0.05, Figure 7E). For GIN, we found miR-26a-5p-inhibitor increased the level of γ-H2AX about 1.8-fold (*p* < 0.001, Figure 7F), and increased the micronuclear counts about 1.8-fold (*p* < 0.001, Figure 7F). In order to verify the role of MTDH in OTUD6B-AS1/miR-26a-5p pathway, we performed following experiments. As Figure 7G showed, down-regulation of MTDH significantly decreased the LC3B-II focus counts about 50% (*p* < 0.001, Figure 7G). And the WB assay displayed that down-regulation of MTDH decreased the level of LC3B-II and the ratio of LC3B-II/LC3B-I about 50% (*p* < 0.0001, Figure 7H), while the restorage of MTDH increased the expression of LC3B-II (Figure 7H). Well, MTDH also played a role in GIN. As the Figure 7I showed, down-regulation of MTDH decreased the level of γ-H2AX about 50% (*p* < 0.0001, Figure 7I), and decreased the micronuclear counts more than 50% (*p* < 0.001, Figure 7I). Finally, we explored the roles of up-stream regulator and down-stream target in regulation of DDR process. As the results showed, over-expression of OTUD6B-AS1 down-regulated the level of phosphorylated ATR (p-ATR), ATM and RAD51, and decreased the ratio of p-ATR/ATR, p-ATM/ATM, and p-RAD51/RAD51 (*p* < 0.01, Figure 7J). As for MTDH, we found down-regulation of MTDH increased the level of p-RAD51 and p-ATM, and increased the ratio of p-RAD51/RAD51 and p-ATM/ATM (*p* < 0.01, Figure 7K).

## DISCUSSION

At present, TNBC is still a malignant and refractory subtype of breast cancer. Furthermore, chemotherapy resistance increases the difficulty of treatment of it. GIN is a distinctive feature that distinguishes tumors from normal tissues [34]. It is generally believed that the genetic alteration, induced by tumor tissues in the cytotoxic microenvironment, is an important factor in the generation of acquired drug resistance and also an external manifestation of GIN [34]. In fact, the level of genomic alteration is usually up-regulated in TNBC tissues, which means that the level of GIN in TNBC is higher than other types of breast cancer (Supplementary Figure 3B). The worse genomic stability usually accompanies with the worse prognosis, which is also consistent in breast cancer (Supplementary Figure 3A). DDR is an important biological process to maintain genomic stability, and TP53, BRCA1, ATM, and ATR are important roles in the DDR process [3]. When DNA damage occurs, ATM and ATR are activated, which further activate RAD51, followed by phosphorylation to activate H2AX (γ-H2AX), which is recruited to the DNA damage area for DNA repair [35]. In other words, DDR defects can lead to increased genomic instability. In fact, it is reported that DDR deficiency increases the risk of malignant subtypes (luminal-b and TNBC subtypes), improves drug tolerance and radiotherapy tolerance, and increases risk of cancer metastasis in breast cancer [4].

Recent studies have pointed out that autophagy can regulate DDR-associated protein, maintain genomic stability, and thereby inhibit tumor growth. For example, chaperone-associated autophagy (CMA) maintains the stability of the MRN complex by directly or indirectly regulating CHEK1 levels, thereby promoting DDR [6]. In addition, autophagy participates in DDR by regulating the level of SQSTM1, which promotes the non-homologous end binding (NHEJ) [7, 8]. In tumor-related research, previous study suggests that ncRNA can interfere with genomic instability by regulating autophagy, thereby participating in the regulation of tumor growth, drug resistance and invasion. For example: miRNA-20a can promote genomic instability by inhibiting autophagy, and further lead to the occurrence and development of breast cancer. In this way, autophagy seems to promote the DDR process [9]. As we all know, autophagy has been widely reported as an accelerator of chemotherapy resistance in cancer, and DDR-defect-mediated GIN seems to play a key role in chemotherapy resistance. However, there is a paradox. Based on this paradox, autophagy abolishment inhibits chemotherapy resistance, but accompanying with the higher risk of acquired chemotherapy resistance induced by DDR-defect-mediated GIN. In other words, autophagy defects can increase the short-term sensitivity of tumor cells to chemotherapy drugs, but increase the risk of long-term drug resistance caused by GIN; enhanced autophagy enhances chemotherapy resistance, but weakens the GIN-induced long-term chemotherapy resistance. The above discussion seems to provide a reasonable explanation for the failure of autophagy-target treatment in clinical trials, and ncRNA is likely to be responsible for it. In fact, many studies have shown that ncRNA can participate in the regulation of DDR-related genes, and these RNAs are usually also involved in the regulation of autophagy. For example: miR-29 can inhibit autophagy, and can maintain genomic stability by regulating the level of PIK3R1 in breast cancer [36, 37]; miR-96 can inhibit autophagy, and can promote the GIN by regulating the level of RAD51 in breast cancer [38, 39]; miR-182 can inhibit autophagy by regulating mTOR, and can enhance GIN by regulating BRCA1 in breast cancer [40, 41]. It seems that there is heterogeneity in ncRNA regulation of autophagy and GIN. In addition, autophagy is also an inhibitor of DNA repair. Previous studies have shown that the autophagy activator rapamycin promotes the inhibition of ionizing radiation-induced DSBs repair by significantly inhibiting HR and NHEJ in breast cancer [10]. Therefore, if a factor that regulates both autophagy and DDR is found, it may be a new strategy to reduce short-term and long-term chemotherapy resistance by inhibiting autophagy and promoting breast cancer DDR.

In this study, we identified 3 miRNAs related to autophagy and GIN (Figure 2A and 2B), and all of these miRNAs are prognosis-correlated in breast cancer (Figure 2C). Given that these three miRNAs (miR-26a-5p, miR-151a-5p and let-7b-3p) are more abnormally expressed in TNBC, this means these three miRNAs are very important in the chemotherapy resistance of TNBC (Figure 2F). Among these three miRNAs, we found miR-26a-5p was reported to regulate autophagy in colorectal cancer, osteosarcoma, hepatocellular carcinoma, glioma, and laryngeal squamous cell carcinoma by targeting ULK1/2, DAPK1, and ATG12. And the data analysis of this study showed that the expression level of miR-26a-5p was negatively correlated with the level of GIN (Figure 3D). Basing on the low expression level of miR-26a-5p in breast cancer tissues, especially in TNBC (Figure 2D and 2E), we guess miR-26a-5p may play an important role in PTX resistance. In fact, our study showed that miR-26a-5p-mimic reduced the chemotherapy sensitivity of HCC1937 to PTX, while miR-26a-5p- inhibitor increased the chemotherapy resistance of HCC-1937 to PTX (Figure 6). In addition, we found that when screening PTX-resistant cell lines, miR-26a-5p-inhibitor promoted the formation of PTX resistance, which was more powerful than RAPA treatment. (Figure 6B). This means miR-26a-5p hold other ways to regulate PTX resistance in TNBC. In following experiments, we found miR-26a-5p-inhibitor increased the level of autophagy, significantly up-regulated the level of γ-H2AX, and increased the micronuclear counts (Figure 7C). This means that miR-26a-5p participates in autophagy and DDR to regulate PTX resistance in TNBC. Therefore, we believe that miR-26a-5p interferes with short-term and long-term PTX resistance by regulating autophagy and DDR.

In order to understand the entire pathway of miR-26a-5p regulating PTX resistance, we analyzed its upstream and downstream. In our research, we found lncRNA OTUD6B-AS1 was co-expressed with miR-26a-5p, and MTDH is the common target of OTUD6B-AS1 and miR-26a-5p. On the one hand, studies have shown that OTUD6B-AS1 can inhibit tumor growth in kidney cancer, thyroid cancer, and colorectal cancer, while promote tumor growth in hepatocellular carcinoma [42–45]. However, its effect on breast cancer is unclear. On the other hand, it has been reported that the overexpression of MTDH promotes PTX resistance, tamoxifen resistance and doxorubicin resistance in luminal-A and TNBC breast cancer. MTDH-based DNA vaccines suppress the lungs-metastasis of breast cancer, and enhance the chemotherapy sensitivity. Therefore, OTUD6B-AS1-miR-26a-5p-MTDH may be a new signaling pathway to regulate PTX resistance in TNBC. Excitingly, we found that OTUD6B-AS1 can down-regulate the expression level of miR-26a-5p, but up-regulate the level of MTDH by experiments (Figure 4). At the same time, miR-26a-5p-mimic can down-regulate the level of MTDH (Figure 4). In addition, luciferase analysis showed that OTUD6B-AS1 can interact with miR-26a-5p (Figure 4). Therefore, OTUD6B-AS1-miR-26a-5p-MTDH is indeed an existing regulatory pathway. In the following exploration, by *in vivo* and *in vitro* experiments, we found that down-regulation of MTDH promoted PTX-mediated cytotoxicity (Figure 6F, 6G and 6I). At the same time, by IF and WB experiments, down-regulation of MTDH inhibited PTX-mediated autophagy, and the restorage of MTDH restores PTX-mediated autophagy (Figure 7H). In addition, down-regulation of MTDH significantly reduced γ-H2AX levels and the micronuclei counts, which accompanying with up-regulated activation DDR process (up-regulation of phosphorylated ATM and RAD51 levels). As an upstream regulator, we found that down-regulation of OTUD6B-AS1 inhibited PTX-induced autophagy, inhibited the activation of DDR process (down-regulation of phosphorylation ATM, ATR and RAD51), and promoted GIN (up-regulation of γ-H2AX and micronuclei counts).

So, we believe that OTUD6B-AS1/miR-26a-5p/MTDH promotes the development of paclitaxel resistance in TNBC by inhibition of DNA repair (up-regulates genomic instability) and promotion of autophagy.

## CONCLUSIONS

In general, our study identified OTUD6B-AS1/miR-26a-5p/MTDH was an important signaling pathway in PTX resistance in TNBC, which was not reported before. And in this paper, we verified that OTDU6B-AS1 maintained expression of MTDH by down-regulation of miR-26a-5p. Basing on cytological and biomolecular experiments, we found OTUD6B-AS1/miR-26a-5p/MTDH promoted PTX resistance formation through up-regulation of autophagy and DDR-inhibition-mediated genomic instability. As for the mechanism of MTDH in regulation autophagy and DDR-associated protein activation, we did not provide evidence here, it will be our following work.

## Supplementary Materials

Supplementary Figures

Supplementary Tables
