# Supplementary material for: LncRNA OTUD6B-AS1 promotes paclitaxel resistance in triple negative breast cancer by regulation of miR-26a-5p/MTDH pathway-mediated autophagy and genomic instability
Source: Aging (Albany NY). 2021 Nov 5;13(21):24171–91. doi: 10.18632/aging.203672 (PMC8610138; doi:10.18632/aging.203672)
Supplement: Supplementary Figures [file aging-13-203672-s001.pdf]

## SUPPLEMENTARY FIGURES

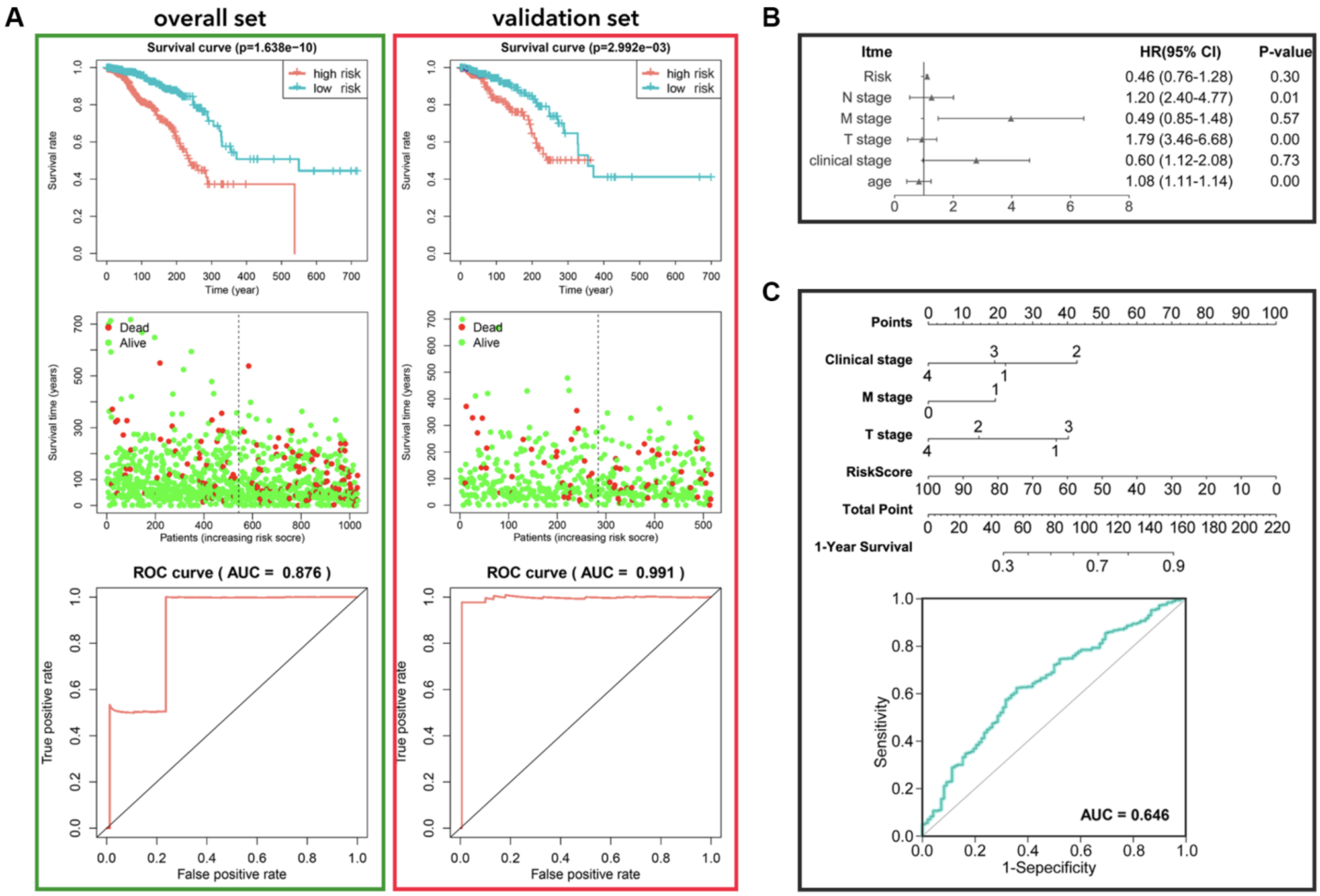


**Supplementary Figure 1. The predict model of prognosis in breast cancer.** (**A**) Breast samples from TCGA were divided into training group and validation group. 22 identified miRNA were analyzed to establish prognosis predict model. (**B**) Multivariate logistics regression analysis screened the independent risk factors. (**C**) Establishing a nomogram by R, and the ROC value was 0.646.

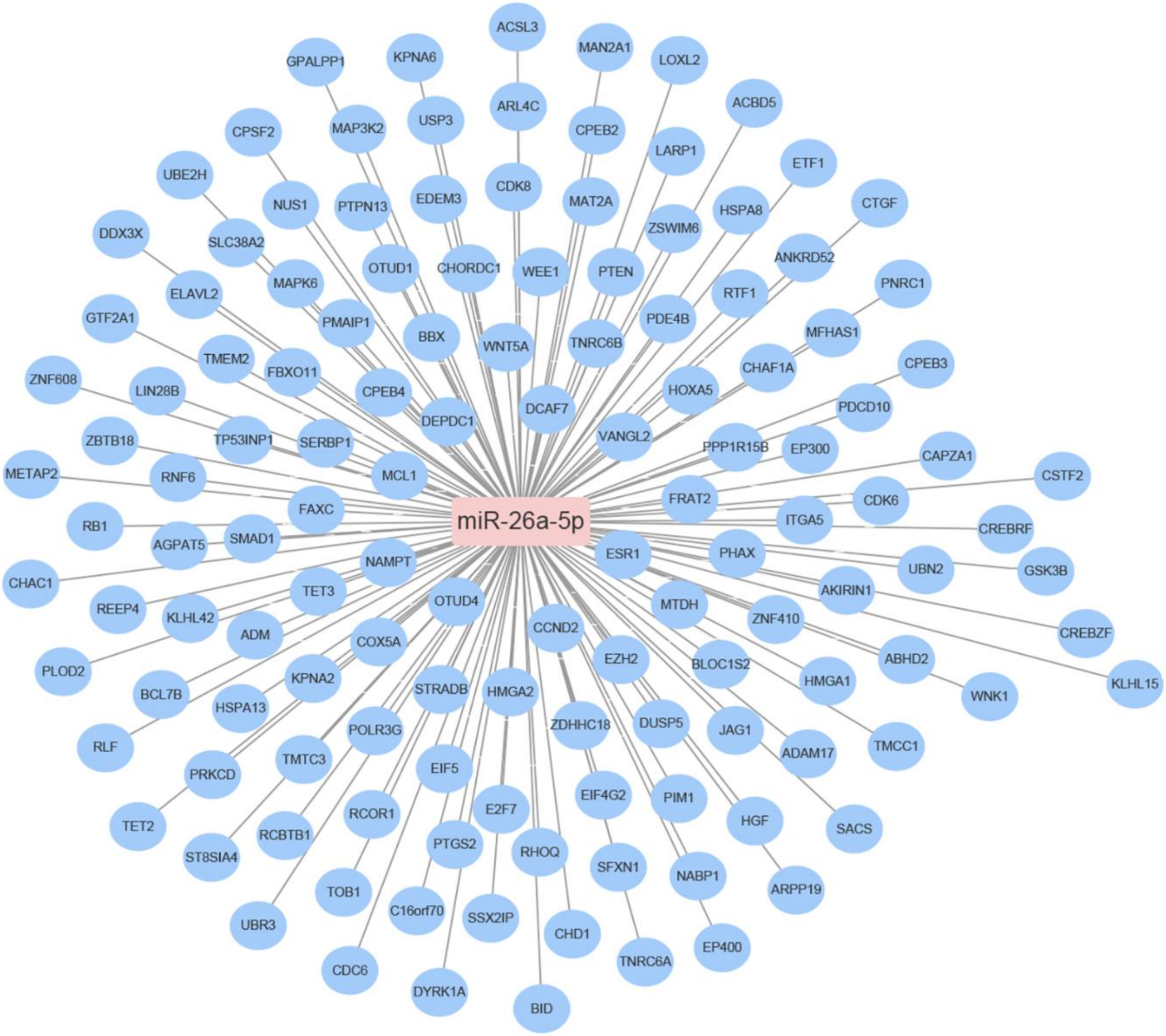


**Supplementary Figure 2. The targeted genes of miR-26a-5p.** 133 targeted genes of miR-26a-5p were identified by miRDB, miRTarBase and TargetScan database.

A

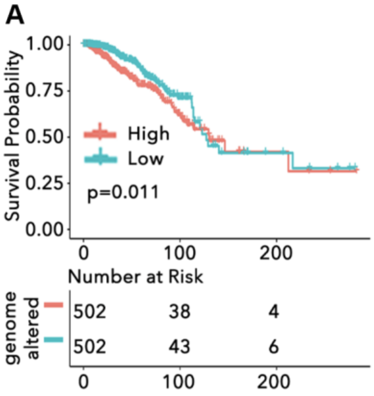


| let-7b-3p | | |
|---|---|---|
| DDR | R-value | p-value |
| PPP4C | -0.2334 | 3.40E-14 |
| SEM1 | -0.2297 | 8.69E-14 |
| POLD1 | -0.2247 | 3.05E-13 |
| RECQL4 | -0.2216 | 6.56E-13 |
| RFC2 | -0.2177 | 1.66E-12 |
| MSH3 | 0.2342 | 2.75E-14 |
| RAD50 | 0.2527 | 1.89E-16 |
| POLR2A | 0.2551 | 9.40E-17 |
| XPA | 0.2667 | 3.25E-18 |
| CCNH | 0.2826 | 2.39E-20 |

59 negative co-expression
36 positive co-expression

| miR-26a-5p | | |
|---|---|---|
| DDR | R-value | p-value |
| POLM | 0.2556 | 8.30E-17 |
| POLL | 0.2636 | 8.10E-18 |
| PARP3 | 0.2723 | 5.92E-19 |
| NEIL1 | 0.3143 | 5.09E-25 |
| RAD51 | -0.2682 | 2.05E-18 |
| MSH6 | -0.2646 | 6.12E-18 |
| NEIL3 | -0.2603 | 2.10E-17 |
| TDG | -0.2416 | 3.91E-15 |
| XRCC5 | -0.2384 | 9.12E-15 |
| PRKDC | -0.2355 | 1.95E-14 |

69 negative co-expression
26 positive co-expression

B

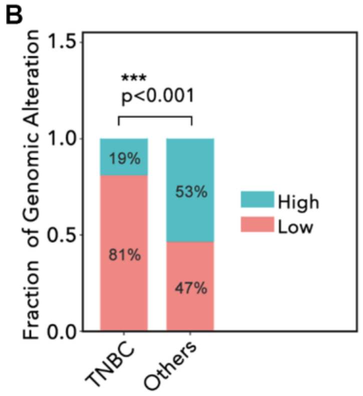


| miR-151a-5p | | |
|---|---|---|
| DDR | R-value | p-value |
| PARP3 | -0.3221 | 2.85E-26 |
| PCNA | 0.3268 | 4.89E-27 |
| NEIL3 | 0.338 | 6.52E-29 |
| PRKDC | 0.3474 | 1.45E-30 |
| POLR2K | 0.3724 | 3.31E-35 |
| RAD51 | 0.3769 | 4.42E-36 |
| RAD54B | 0.378 | 2.75E-36 |
| ELOC | 0.3951 | 8.67E-40 |
| RAD54L | 0.4104 | 4.53E-43 |
| RECQL4 | 0.4313 | 7.11E-48 |

20 negative co-expression
85 positive co-expression

**Supplementary Figure 3. The effect of genomic instability in breast cancer.** (**A**) K–M analysis of prognosis grouped by genomic instability. (**B**) The genomic instability of subtypes of breast cancer. Displaying DDR-associated genes which were co-expressed with let-7b-3p, miR-151a-5p and miR-26a-5p.
