# Supplementary material for: LncRNA OTUD6B-AS1 promotes paclitaxel resistance in triple negative breast cancer by regulation of miR-26a-5p/MTDH pathway-mediated autophagy and genomic instability
Source: Aging (Albany NY). 2021 Nov 5;13(21):24171–91. doi: 10.18632/aging.203672 (PMC8610138; doi:10.18632/aging.203672)
Supplement: Supplementary Tables [file aging-13-203672-s002.pdf]

SUPPLEMENTARY TABLES

Supplementary Table 1. Sequences of miR-mimic, miR-inhibitor and siRNA.

mimic sequences of miRNAs	Sequence 5′ to 3′
miR-26a-5p	5′ UUCAAGUAAUCCAGGAUAGGCU 3′
miR-151a-5p	5′ UCGAGGAGCUCACAGUCUAGU 3′
let-7b-3p	5′ CUAUACAACCUACUGCCUUCCC 3′
**Inhibitors sequences of miRNAs**	**Sequence 5′ to 3′**
miR-26a-5p	5′ AGCCUAUCCUGGAUUACUUGAA 3′
miR-151a-5p	5′ ACUAGACUGUGAGCUCCUCGA 3′
let-7b-3p	5′ GGGAAGGCAGUAGGUUGUAUAG 3′
Negative control	5′ UUCUCCGAACGUGUCACGUTT 3′
miR-inhibitor N.C	5′ CAGUACUUUUGUGUAGUACAA 3′
**siRNAs of lncRNA and MTDH**	**Sequence 5′ to 3′**
si-OTUD6B-AS1	5′ CCAGTGCCATCAACCTCATACGTAT 3′
si-MTDH	5′ CATTGCTGCTTGGTCTAGTGTGGAT 3′

Supplementary Table 2. The prognosis-related miRNAs in breast cancer.

miRNA	*p*-value	
	K-M analysis	Univariate cox regression
hsa-let-7b-3p	0.02113980	0.03047540
hsa-let-7b-5p	0.01473512	0.01275281
hsa-miR-1307-3p	0.00447645	0.01934154
hsa-miR-146a-5p	0.04568606	0.04061888
hsa-miR-148b-5p	0.00469098	0.00323225
hsa-miR-150-5p	0.00391739	0.03947643
hsa-miR-151a-5p	0.01116057	0.02742938
hsa-miR-185-5p	0.04742121	0.02550837
hsa-miR-205-3p	0.03652528	0.04677589
hsa-miR-26a-5p	0.03318323	0.04082386
hsa-miR-26b-3p	0.04795272	0.03483932
hsa-miR-30c-1-3p	0.00014685	0.00390520
hsa-miR-340-3p	0.00600939	0.00713545
hsa-miR-340-5p	0.02429416	0.00179968
hsa-miR-363-3p	0.04724814	0.03780897
hsa-miR-3926	0.02632386	0.03167946
hsa-miR-4772-3p	0.00300019	0.01257781
hsa-miR-556-5p	0.02525752	0.01676248
hsa-miR-627-5p	0.00551633	0.02032374
hsa-miR-7706	0.0150763	0.01736402
hsa-miR-9-3p	0.03233505	0.01274037
hsa-miR-99b-3p	0.04958775	0.00948401
